# Fertility-Sparing Approaches in Atypical Endometrial Hyperplasia and Endometrial Cancer Patients: Current Evidence and Future Directions

**DOI:** 10.3390/ijms23052531

**Published:** 2022-02-25

**Authors:** Nayanar-Adela Contreras, Jordi Sabadell, Paula Verdaguer, Carla Julià, Maria-Eulalia Fernández-Montolí

**Affiliations:** 1 Gynecology Department, Hospital El Pilar, Balmes 268-270, 08006 Barcelona, Spain; nayanarcp@gmail.com; 2 Department of Gynaecology, Hospital Universitari Vall d’Hebron, Vall d’Hebron Barcelona Hospital Campus, Universitat Autònoma de Barcelona, Passeig Vall d’Hebron 119-129, 08035 Barcelona, Spain; jsabadell@vhebron.net; 3 Department of Gynaecology-ASSIR, Ronda General Prim 35, Mataró, 08302 Barcelona, Spain; pvm0368@gmail.com; 4 Department of Gynaecology, Hospital de Viladecans, Avda de Gavà 38, Viladecans, 08840 Barcelona, Spain; cjuliahv@gencat.cat; 5 Department of Gynaecology, Hospital Universitari de Bellvitge, Universitat de Barcelona-IDIBELL, Feixa Llarga s/n, L´Hospitalet de LLobregat, 08907 Barcelona, Spain

**Keywords:** endometrial neoplasms, endometrial hyperplasia, fertility preservation, progestins, organ sparing treatments, meta-analysis, randomized clinical trials

## Abstract

Endometrial cancer (EC) is the fourth most common cancer in women in developed countries. Although it is usually diagnosed in postmenopausal women, its incidence has increased in young women, as well in recent decades, with an estimated rate of 4% in those under 40 years of age. Factors involved in this increase, particularly in resource-rich countries, include delayed childbearing and the rise in obesity. The new molecular classification of EC should help to personalize treatment, through appropriate candidate selection. With the currently available evidence, the use of oral progestin either alone or in combination with other drugs such as metformin, levonorgestrel-releasing intrauterine devices and hysteroscopic resection, seems to be feasible and safe in women with early-stage EC limited to the endometrium. However, there is a lack of high-quality evidence of the efficacy and safety of conservative management in EC. Randomized clinical trials in younger women and obese patients are currently underway.

## 1. Introduction

Endometrial cancer (EC) is the fifth most common cancer in women. Its incidence is increasing in high-income countries, where it is now the fourth most prevalent cancer in women [1,2]. Over 300,000 new cases of endometrial cancer are diagnosed annually worldwide; recent annual figures were 9703 in the UK and 66,570 in the US (2018) [3,4]. The incidence rates in 2020 were estimated to be 21.4 per 100,000 in North America, 16.6 per 100,000 in Europe as a whole, and 13.1 per 100,000 in Spain, attributable to the greater overall prevalence of obesity and metabolic syndromes [5,6,7].

Endometrial hyperplasia is considered a precursor of certain types of endometrial cancer. In the 2014 World Health Organization (WHO) classification, endometrial hyperplasia includes hyperplasia with and without atypia [8].

Progression rates to cancer of 1–5% have been reported for hyperplasia without atypia, and of nearly 25% for hyperplasia with atypia [9]. Kurman et al. [10] estimated the risk of progression of endometrial hyperplasia to carcinoma to be between 1% and 3% during a mean follow-up of 13 years. More recently, Lacey et al. reported a risk of progression of below 5% (including simple hyperplasia and complex hyperplasia) after diagnosis of endometrial hyperplasia over a 20-year follow-up [11].

EC is commonly observed in postmenopausal women, but between 15% and 25% of cases are found pre-menopause [12,13]. It has been estimated that 4% of cases occur in women under the age of 40 years [13,14].

Most endometrial cancer cases are sporadic, with only 3% being associated with Lynch Syndrome [15]. Endometrial carcinoma has been categorized into two pathological types: Type I and Type II. Type I, the endometrioid type, is estrogen-dependent, associated with obesity, polycystic ovary syndrome (PCOS) and a genetic predisposition, and represents approximately 85% of endometrial cancer. Type II comprises the non-endometrioid subtypes that include serous, clear-cell, undifferentiated carcinomas and malignant mixed Mullerian tumors, and are associated with higher patient age, high stage and grade, and poor prognosis [16].

Many factors are associated with the development of endometrial cancer. The main risk factor is obesity because it is associated with peripheral estrogen conversion via aromatization in adipose tissue [17]. Other risk factors are: hyperinsulinemia, diabetes, hypertension, nulliparity, anovulatory cycles and sedentary lifestyle [14,16]. Women taking tamoxifen should be informed about the risks of endometrial proliferation, hyperplasia and cancer. They should be encouraged to consult their gynecologist in case of any abnormal vaginal bleeding [18,19].

The standard treatment for EC is hysterectomy and bilateral salpingo-oophorectomy (THBSO) with or without lymphadenectomy [19,20,21,22]. Survival outcomes are good, from 74% to 91%, particularly for low-grade endometrioid tumors without lymph node involvement [14,20,22].

Fertility-sparing treatment involves pharmacological and non-pharmacological interventions. The most frequently used are oral progestin, medroxyprogesterone acetate (MPA) or megestrol acetate (MA) [19,21,22]; other treatments are gonadotropin-releasing hormone agonists (GnRHa), levonorgestrel-releasing intrauterine system (LNG-IUS) [19,21,22], and metformin plus progestin. Recently, other strategies have been included, such as hysteroscopy resection followed by hormonal therapy [22] or bariatric surgery as a weight loss strategy.

Today, a very high percentage of the European population delay childbearing, a practice that has led to an increasing number of nulliparous women at the time of diagnosis of endometrial cancer. In the US, pregnancy rates increased in women aged >30 from 1990 to 2015 [23]. In this scenario, it is very important that women with a diagnosis of gynecological malignancy should seek counseling regarding fertility preservation options. Optimal selection of candidates for this treatment is necessary and, as soon as the disease remits, they must be referred to assisted reproductive techniques in order to achieve pregnancy [24].

## 2. Criteria for Fertility-Sparing Treatment in Endometrial Cancer

The selection of patients with EC for fertility-sparing treatment is important in order to achieve the best outcomes. Current guidelines recommend progestin therapy in stage IA, grade 1 (well-differentiated) endometrioid-type endometrial cancer [24,25].

In 2015, Rodolakis et al. published guidelines for gynecological oncologists treating women with EC who wish to preserve their fertility. Finally, a consensus was reached between the ESMO, the European Society of Gynecological Oncology, and the European Society for Radiotherapy & Oncology to help identify the ideal candidates for this conservative treatment. The most important points to bear in mind are: first, the assessment of the tumor, including histological type, grade, myometrial invasion, and presence of lymphovascular space invasion; second, the treatment offered, the type, dose, duration of medical intervention, and follow-up [26].

An update published in 2017 recommended the following criteria for fertility-sparing management in EC women: grade 1 EC or atypical endometrial hyperplasia (AEH), histological diagnosis using dilatation and curettage with or without hysteroscopy, and myometrial invasion. Extrauterine disease should be ruled out using pelvic magnetic resonance imaging (MRI) or expert transvaginal ultrasound (TVUS) [27].

The 2018 National Comprehensive Cancer Network (NCCN) includes the following criteria to consider fertility-sparing management of endometrial cancer: well-differentiated (grade 1) endometrioid endometrial carcinoma [19]; disease thought to be limited to the endometrium on magnetic resonance imaging (preferred) or ultrasound; absence of suspicious or metastatic disease on imaging [19,25,26,27]. Table 1 shows the optimal indications for fertility-sparing treatment. 

### 2.1. Histologic Diagnosis

Dilatation and curettage biopsy is recommended for the histologic diagnosis of endometrial cancer. Hysteroscopic biopsy is also a precise diagnostic method for endometrial adenocarcinoma [26]. However, the most accurate sampling method has not yet been established. Review by more than one pathologist or by a pathologist specializing in gynecological cancers should be considered [17,27].

### 2.2. Determination of Extent of the Disease

Myometrial invasion is another important prognostic factor in patients with endometrial cancer. According to the Fédération Internationale de Gynécologie et d’Obstétrique (FIGO) 26th Annual Report, the 5-year overall survival rate in patients with tumors limited to the endometrium is as high as 90.8%; however, this rate drops to 85.4% when deep myometrial invasion is identified [17].

In the evaluation of myometrial invasion, contrast-enhanced MRI is the preferred method as it offers higher accuracy than TVUS [17,19,21,27].

Recently, the utility of positron emission tomogram (PET) in the detection of lymph node metastases in early-stage endometrial cancer cases has been assessed, with a sensitivity of 63% and a specificity of 94.7% [28]. In a study of 53 patients, Park et al. compared the use of PET and MRI in the preoperative study of patients with EC for detection of primary lesions and lymph node (LN) and distant metastases. They concluded that PET had moderate sensitivity for LN metastases and could not replace surgical staging; for distant metastases, however, it had a sensitivity of 100% and a specificity of 93.8%, making it an interesting alternative in patients who are poor candidates for surgery [29].

Fertility-sparing treatment of grade 2 endometrioid endometrial cancer is reported in very limited case studies and should only be considered in highly selected individuals with a shared decision-making approach [24]. The most recent publications in this regard are discussed in the text below.

There must not be any contraindications to medical therapy or pregnancy. Patients must be informed that the fertility-sparing option is not the standard of care for the treatment of endometrial carcinoma [19,25].

## 3. Molecular Classification in Endometrial Cancer

In 2013, a molecular diagnostic classification published by The Cancer Genome Atlas (TCGA) Research Network defined four prognostic categories: POLE ultra-mutated, microsatellite instability hypermutated, low copy-number tumor, and high copy-number tumor [30,31,32]. It is now known that low-grade Endometrioid Endometrial Carcinoma have distinctive molecular features. Classically, EC categorized in Type I (endometrioid endometrial cancer), presents a PTEN mutation, and Type II (serous endometrial cancers) presents a p53 mutation. Nevertheless, epigenetic cell modifications are gaining importance in cancer etiopathogenesis and characterization [30]. Table 2 displays the main molecular aspects and medical applications of the four molecular types which will be the basis of the new classification presented below [32,33].

In 2019, Britton et al. published a study of the prognostic significance of Proactive Molecular Risk Classifier for Endometrial Carcinoma (ProMisE) in young (<50 years) women with EC. The ProMisE molecular classifier can be applied to endometrial biopsy, demonstrating high concordance with final hysterectomy in this series Ƙ = 0.87, consistent with the literature. It uses pragmatic molecular tests to identify ECs with mismatch repair deficiency (MMRd), mutations in the exonuclease domain of DNA polymerase epsilon (POLE), and wild type or aberrant p53 expression (p53 wt or p53 abn respectively) [34].

## 4. Pharmacological and Non-Pharmacological Interventions

The current therapeutic approach to endometrial cancer and atypical endometrial hyperplasia is based on the use of oral progestins such as medroxyprogesterone acetate, megestrol acetate or gonadotropin-releasing hormone agonists [16,24,35] or levonorgestrel-releasing intrauterine system [19,36,37]. A few studies have assessed the effectiveness of adding metformin [32]. Current recommendations are MPA at a dose of 400–600 mg/day or MA at a dose of 160–320 mg/day for a minimum of six months, with follow-up assessment using biopsy and imaging [28,36,37]. Levonorgestrel-IUS releases 52 mg of intrauterine progestin for up to five years.

The goal of hormone treatment is to counterbalance the action of estrogen. Progestogens have antiproliferative actions such as estrogen receptor (ER) and insulin-like growth factor 1 (IGF-1) inhibition and antiangiogenic action [38]. Low-grade endometrial cancer often presents estrogen and progesterone receptors (PR). PR-negative endometrial cancer does not respond to therapy with progestogens [39,40,41]. Progesterone treatment for EC has achieved complete response rates of 55–76% and recurrence rates of 20–47% [42,43,44,45].

Several randomized controlled trials (RCTs) are currently underway to evaluate the efficacy of LNG-IUS with or without oral progestin or metformin in younger women with low-grade EC [46,47,48].

Metformin shows several antiproliferative mechanisms: decrease in insulin and IGF-1 levels, increase in progesterone receptor concentration and reduction in progesterone resistance [49,50]. The results of a meta-analysis concluded that metformin could reverse the proliferation biomarkers associated with tumor progression, and could contribute to improving survival after endometrial cancer [49]. Other authors, such as Acosta-Torres et al., found no difference in complete response (CR) when adding metformin to progestogens; however, the live-birth or pregnancy rate did not rise above 20%, and most patients required assisted reproductive technology (ART) [51].

Pharmacological interventions, such as appetite suppressants or drugs to reduce fat absorption, may be used to promote weight loss. In obese women, weight-loss interventions (lifestyle interventions) achieved changes in blood biomarkers associated with this cancer [49]. A meta-analysis showed that increased body mass index (BMI) was associated with increased mortality in endometrial cancer [52]. Weight-loss interventions may help to improve survival in EC patients through pathways that connect obesity and endometrial cancer [52,53,54].

A review of bariatric surgery treatment for EC prevention concluded that this approach seems to reduce the risk of endometrial cancer, but these results need to be reproduced in randomized clinical trials [55]. Bariatric surgery can lower glucose levels and insulin and insulin-like growth factor-binding protein 1 (IGFBP-1); in addition, this surgery can improve insulin sensitivity in obese women and decreases inflammatory endometrial cancer risk biomarkers [56].

In addition to weight reduction, there are reports of high rates of improvement, and even cure, of comorbidities associated with obesity, including type II diabetes mellitus, obstructive sleep apnea, hypertension, asthma, osteoarthritis, risk of cancer and gastro-esophageal reflux disease [57,58]. A meta-analysis demonstrated that the risk of endometrial cancer rises as weight increases [59]. In obese women, there is a metabolic state that promotes oncogenesis; this state is related to hyperoestrogenemia, inflammation and insulin resistance, and leads to multiple changes in oncogenic signaling pathways [60]. These findings have identified potential targets for treatment.

Hysteroscopy, as part of a conservative approach to endometrial cancer or atypical endometrial hyperplasia, is controversial. Some authors restrict its use to diagnosis of the tumor and assessment of margins; others explore the possibility of primary treatment plus progestin orally or LNG-IUS, and report excellent complete response rates. Alonso et al. [61] reviewed 39 years of published studies of young patients with early stage 1A of EC treated with initial hysteroscopic resection followed by fertility-sparing hormone therapy. The inclusion criteria were met by six studies, mostly case series, and a total of 30 patients were included in the statistical analysis. The result shows a complete response rate of 88.9%, and a pregnancy rate of 25% which rose to 66% when ART was applied.

Recent studies have confirmed these findings, reporting complete response rates in women with stage 1A EC of 89% to 97% following hysteroscopic fertility-sparing treatment, and pregnancy rates of over 45% [62,63]. Giampaolini et al. [64] demonstrated that hysteroscopic treatment followed by LNG-IUS had a high efficacy as a fertility-sparing treatment, reporting a response rate of 78.6%.

Concerns have been raised about the possible negative obstetric outcomes due to mechanical damage of the endometrium, causing Asherman’s syndrome and raising the risk of placental accretism [65]. More studies are necessary to provide further support for the hysteroscopy plus hormone treatment as routine clinical treatment.

## 5. Current Evidence on Fertility-Sparing Treatments for Endometrial Cancer and Atypical Endometrial Hyperplasia

In general, fertility-sparing management of endometrial cancer is associated with acceptable rates of progression free-survival (PFS) and overall survival (OS). In a cohort study including 6339 women with endometrial cancer, 161 (2.5%) initially received hormone therapy (HT), and long-term survival for young patients with Grade 1 EC was above 90% after both 10 and 15 years. Initial analyses suggested a higher endometrial cancer-specific 15-year mortality rate in patients treated with HT than in the primary surgery group. The hazard ratio for OS was 1.45 (95% CI, 0-44-4.74) [66]. Further discussion is warranted of the optimal approach to follow-up, without compromising patients’ quality of life or increasing the risks associated with recurrence or survival [67].

In 2021, Fernández-Montoli et al. [68] published a systematic review protocol for fertility-sparing treatment for atypical endometrial hyperplasia and EC. This review will help to clarify the effectiveness and risks of fertility-preserving treatments, including pathologic complete response rate, live birth rates, progression of disease and need for surgical treatment (i.e., hysterectomy).

Table 3 summarizes the systematic reviews and meta-analyses of fertility-sparing treatments in patients with early endometrial cancer (EEC), and the objectives evaluating the oncologic and reproductive outcomes.

Gallos et al. [43] published a meta-analysis, including observational studies of EEC and complex atypical hyperplasia (CAH). The objective was to evaluate the regression, relapse and live birth rates. The meta-analysis included 38 studies with 408 cases of EEC and 151 of CAH. Twenty cases of concurrent ovarian cancer were reported, ten of which progressed and two died during the follow-up. It seems that relapse may be more likely for obese women, but the conclusion is that fertility-sparing treatment of EC and CAH is feasible.

Baker et al. [48] published a systematic review of CAH and EEC with oral progestin or (LNG-IUS), including only patients with more than six months of treatment. The review comprised 12 studies with 219 women using oral progestin and 11 LNG-IUS, 117 cases of CAH and 102 of EEC. The progression from CAH to EC observed was 2.7%. The available evidence suggests that treatment with oral or intrauterine progestin is equally effective.

Koskas et al. [69] in 2014 published a systematic review to evaluate various possible prognostic factors for the fertility-sparing management of atypical hyperplasia and endometrial cancer. The review comprised 24 studies with 370 women. In the multivariate analysis, previous pregnancy, infertility and treatment with megestrol acetate were associated with a higher remission rate. The regression rate was 78% in 12 months and 81% in 24 months, and the global progression rate was 15%. They concluded that fertility-sparing management was not contraindicated in older patients with previous infertility or obesity.

In 2017, Wei et al. [45] compared the different strategies for fertility preservation, in a meta-analysis with EEC and CAH including 28 studies with 1038 women. The aim was to compare them by evaluating oncologic and reproductive outcomes. In recent years, LNG-IUS and oral progestin plus LNG-IUS have emerged as treatment options. In patients with EEC and CAH, treatments with progestin, and with or without LNG-IUS can achieve high complete response rates; however, the pregnancy outcomes may be worse in patients treated with LNG-IUS alone.

Luo et al. [70] published a Cochrane review of the efficacy of oral progestin and LNG-IUS in patients with atypical endometrial hyperplasia. RCTs of oral progestin and LNG-IUS versus other treatments or placebo were included. Only 19 women with atypical complex hyperplasia met the selection criteria, and so the quality of evidence for determining a difference in regression rate between treatments was very low. The authors concluded that large studies are needed to assess the efficacy and safety of oral progestin and LNG-IUS to treat atypical endometrial hyperplasia.

Fan et al. [71] performed a review about the efficacy of different treatments in preserving fertility for grade 1 presumed stage IA endometrial cancer, including 28 studies with 619 women. They divided the analysis of the results into three treatment groups: oral progestin alone, hysteroscopic resection plus progestin therapy, and LNG-IUS plus gonadotropin-releasing hormone agonists/progestin therapy. The study concluded that patients who received hysteroscopic resection followed by progestin therapy achieved higher complete response rate.

Guillon et al. [72] published a systematic review aimed to identify remission rates and prognostic factors in patients with endometrial cancer and atypical hyperplasia undergoing fertility preservation management. It included 65 studies with 1604 women, and analyzed three types of prognostic factors associated with remission rate: patient characteristics, management characteristics, and study characteristics. The authors concluded that the use of hysteroscopy as a sampling method and a higher ratio of infertile patients were prognostic factors associated with a higher remission rate.

Chae-Kim et al. [73] recently published a systematic review of progestin therapy combined with metformin for atypical endometrial hyperplasia or early-stage endometrial cancer in reproductive-aged women. The review included six studies with 621 women, 241 treated with progestin + metformin and 380 treated with progestin only. The authors concluded that progestin plus metformin therapy compared with oral progestin alone achieved lower relapse rates but similar remission and clinical pregnancy rates.

The results of these systematic reviews show that fertility-sparing management is possible and safe in patients with atypical endometrial hyperplasia and early-stage endometrial cancer, with complete responses around 75% for oral progestin and 79% for LNG-IUS. Recurrence rate is around 33% for oral progestin but lower with other treatments such as LNG-IUS (11%) and hysteroscopic resection (14%). However, the live birth rate remains low, around 20–48%, despite the various strategies used.

## 6. Ongoing Studies

Several prospective studies and randomized controlled trials are underway to elucidate the best therapeutic option for patients with endometrial cancer who wish to preserve fertility. These studies should provide us with valuable results for the treatment of these patients, improve survival, reduce relapses, and obtain better obstetric outcomes. Table 4 describes the main ongoing trials, which are yet to be published at the time of writing.

Thirteen clinical trials are registered in clinicaltrials.gov of fertility preservation in patients with early-stage endometrial cancer and atypical hyperplasia. Here, we present a short description of these studies; Table 4 displays the main characteristics of each one.

Clinical Trial NCT00788671: A phase II trial studying the efficacy of the levonorgestrel-releasing intrauterine system in treating patients with complex atypical hyperplasia or grade I endometrial cancer.

Clinical Trial NCT02335203, comparing pre- and post-treatment glandular cellularity in women with complex atypical hyperplasia or grade 1–2 endometrial adenocarcinoma who are treated with intramuscular depot medroxyprogesterone acetate (DMPA) versus placebo injection prior to hysterectomy.

Clinical Trial NCT02342730, a pilot clinical trial studying weight loss referral for healthier survivorship in obese stage I–II endometrial cancer patients or patients with atypical hyperplasia.

Clinical Trial NCT02397083, a randomized phase II trial studying the efficacy of the levonorgestrel-releasing intrauterine system alone or with everolimus in patients with atypical hyperplasia or stage IA grade 1 endometrial cancer.

Clinical Trial NCT02990728, studying the efficacy of LNG-IUS with or without metformin, as fertility-preserving treatment for grade 1 endometrioid adenocarcinoma of the endometrium.

Clinical Trial NCT03042897, a pilot clinical trial studying exercise and diet intervention in promoting weight loss in obese patients with stage I endometrial cancer.

Clinical Trial NCT03241914, a randomized study of the effectiveness of megestrol acetate plus LNG-IUS, aiming to demonstrate that it is not inferior to megestrol acetate alone for returning the endometrial tissue to a normal state in patients with early endometrial cancer.

Clinical Trial NCT03463252, analyzing the effectiveness of LNG-IUS, in the fertility-sparing treatment of atypical endometrial hyperplasia and early endometrial carcinoma, including pathology response and pregnancy outcome.

Clinical Trial NCT04008563 (Bi-FiERCE), a novel study that combines a surgical treatment (bariatric surgery) with the classic treatment of progestogens in patients with atypical hyperplasia and grade 1 cancer of the endometrium.

Clinical Trial NCT04046185, a randomized controlled trial comparing programmed death-1 (PD-1) inhibitor combined with progesterone versus progesterone alone in the treatment of early stage endometrial cancer.

Clinical Trial NCT04362046 (FETCH), evaluating the use of hysteroscopic resection in women diagnosed with atypical endometrial hyperplasia or grade I endometrial cancer who have not responded to hormone therapy.

Recently, the feMMe-controlled trial was published, in which women with EC and AEH treated with LNG-IUS alone or plus metformin (M) or weight loss (WL) intervention. Thirty-five participants were randomized to observation, 36 to WL and 47 to M (10 patients were withdrawn). The results were promising: complete response rate was 82% for AEH and 43% for EC. In addition, the use of weight loss regimen plus LNG-IUS improved the treatment success, achieving an encouraging response rate of 67% [74]. Another study, the FELICIA trial, compared the addition of metformin to medroxyprogesterone acetate for fertility-sparing treatment of AEH and EC [75].

**Table 4:** Ongoing clinical trials of conservative treatment for endometrial cancer (EC).

Clinical Trials ID	Start Date	Study	Aims	Design/ Intervention	Region	Participants
NCT00788671	November 2008	LNG-IUS in patients with complex atypical hyperplasia or Grade I endometrial cancer	- Efficacy of LNG-IUS - Response rate at 1 year	Phase 2 open label trial Levonorgestrel-IUS	USA	70 women 18 years Histology: CAH or EC within 3 months of study enrollment
NCT01686126 (Results on reference [75])	December 2012	Improving the treatment for women with early-stage cancer of the uterus (feMMe)	Pathological complete response	RCT, Open-labelMirena + metformin Mirena alone Mirena + weight loss intervention	Australia	165 women 18 years BMI > 30 kg/m^2^ Histology: CAH or EEC
NCT02335203	January 2015	The effect of neoadjuvant DMPA on glandular cellularity in women awaiting hysterectomy	Change in glandular cellularity	RCT, Open-label Depot medroxyprogesterone acetate	USA	76 women 18 years Histology: CAHG1 or G2 EC Waiting for hysterectomy
NCT02342730	December 2014	Weight loss referral for healthier survivorship in obese stage I-II endometrial cancer survivors or atypical hyperplasia	- Accrual with intervention - Compliance with intervention	Open-label trial Weight loss referral	USA	127 women 18–65 years Histology: Stage I or II EC or CAH BMI > 30 kg/m^2^
NCT02397083	September 2015	Levonorgestrel-releasing intrauterine system with or without everolimus in treatment patients with atypical hyperplasia or stage IA G1 endometrial cancer	Response rate at 3 and 6 months	RCT, Open-label LNG-IUS alone LNG-IUS plus Everolimus	USA	270 patients ≥18 years histology: CAH or grade1 EC or focal grade 2
NCT02990728	March 2016	Mirena^®^ ± metformin as fertility-preserving treatment for young Asian women with early endometrial cancer	Efficacy of Mirena^®^, with or without metformin	RCT, Open-label LNG-IUS alone LNG-IUS + Metformin	Taiwan	120 patients >40 years Histology: G1 ECTumor confined to the endometrial on MRI or TVUS
NCT03042897	February 2017	Exercise and diet intervention in promoting weight loss in obese patients with stage I endometrial cancer	To determine if participants decrease fat mass by 10% after 16 weeks	Interventional, Open-label Supportive Care (exercise and diet)	USA	25 women Histology: stage I EC BMI > 30 kg/m^2^
NCT03241914	August 2017	Megestrol Acetate plus LNG-IUS in young women with early endometrial cancer	- Pathological response rate - Pathological response time	RCT, Open-label Megestrol acetate 160 mg/day Megestrol acetate 160 mg/day plus LNG-IUS for 3 months	China	40 patients 18–45 years Histology: EEC based upon hysteroscopy No myometrial invasion confirmed by MRI
NCT03463252	April 2018	Value of LNG-IUS as fertility-preserving treatment of AEH and EC	- Effectiveness of LNG-IUS - Pathology response - Pregnancy rate	RCT, Open-label trial MPA 250–500 mg/day vs. MPA + LNG-IUS vs. LNG-IUS alone	China	224 patients <40 years Histology: G1 EEC limited to the endometrium by MRI
NCT04008563	August 2020	Bariatric surgery for fertility-sparing treatment of atypical hyperplasia and grade 1 cancer of the endometrium (Bi-FiERCE)	- Recruitment rate—Completion of bariatric surgery - Loss to follow-up rate - Complete response rate	RCT Patients will be randomized 1:1 to Bariatric surgery plus LNG-IUS vs. LNG IUS alone	USA	36 patients 18–41 years Histology: Grade 1 EEC or CAHBMI ≥ 35 kg/m^2^ No evidence of metastatic disease Desire for fertility preservation
NCT04046185	October 2019	Programmed Death-1 (PD-1) Inhibitor combined with progesterone treatment in endometrial cancer	- Pathologic complete remission rate of endometrial curettage tissues - Pathologic partial remission rate of endometrial curettage tissues	RCT Experimental: PD-1 inhibitor and progesterone (toripalimab. 240 mg intravenous injection) + Megestrol Acetate 160 mg/day	China	60 participants Age < 45 years Histology: EEC Grade 1 or Grade 2 Desire to preserve fertility
NCT04362046	April 2020	Fertility sparing management of endometrial cancer and hyperplasia (FETCH)	- Conception rate - Local disease control rate - Distant disease control rate	Prospective, Open-label Hysteroscopic uterine resection for patients who fail progestin therapy	Canada	30 participants Age 19–39 years Histology: Grade 1 EEC or AEH MRI < 1/3 myometrial invasion
NCT04491643	September 2020	Megestrol Acetate plus Rosuvastatin in young women with early endometrial carcinoma	Pathological response rate	Open-label trial Megestrol Acetate 160 mg/day plus Rosuvastatin 10 mg/day	China	43 participants Age 18–45 years Diagnosis based by hysteroscopy of Grade 1 EEC
jRCT2031190065 (Protocol on reference [76])	July 2019	Medroxyprogesterone acetate plus metformin for fertility-sparing treatment of atypical endometrial hyperplasia and endometrial carcinoma (FELICIA trial)	- 3 years relapse-free survival (RFS) - RFS rate - Overall response - Conception rate	RCT, open-label trial MPA alone (600 mg/day) MPA + Metformin (750 mg/day) MPA + Metformin (1500 mg/day)	Japan	120 participants Age 20–42 years Histology: AEH or Grade 1 EEC No prior treatment with high dose progestin Follow up 3 years

Abbreviations: CAH, complex atypical hyperplasia; EC, endometrial cancer; EEC, early endometrial cancer; AEH, atypical endometrial hyperplasia; LNG-IUS, levonorgestrel-releasing intrauterine devices; MA, Megestrol Acetate; MPA, medroxyprogesterone acetate; MRI, magnetic resonance imaging; PD-1, programmed death-1; RCT, randomized controlled trial; TVUS, transvaginal ultrasound; DMPA, depot medroxyprogesterone acetate.

## 7. Fertility-Sparing Treatment for Endometrial Cancer in Special Situations

### 7.1. Grade 2 Endometrial Cancer

Currently, only small retrospective case series including Grade 2 endometrioid endometrial cancer (EEC) for fertility preservation has been published. One reported a similar complete response rate and recurrence rate to Grade 1 EEC [76].

The European Society of Gynecological Oncology (ESGO), the European Society for Radiotherapy and Oncology (ESTRO), and the European Society of Pathology (ESP) have published updates on endometrial cancer. To date, very few studies have been published on the preservation of fertility in stage IA Grade 2 endometrioid carcinoma without myometrial invasion, using MPA + LNG-IUS. The few performed were carried out by specialized gynecologists with a well-designed protocol and correct follow-up [22].

Hwang et al. [77] published a retrospective study of fertility management in Grade 2 patients with stage IA endometrial cancer with combined oral medroxyprogesterone acetate (MPA)/(LNG-IUS) Five patients were included with a mean follow-up period of 44.4 months. The authors concluded that this treatment is effective, but the study is preliminary and a protocol is necessary for these patients.

In 2020, Falcone et al. published a multicenter project endorsed by the Gynecologic Cancer Inter-Group, for patients with G2 endometrioid EC. The study included 23 patients, of whom 74% received hysteroscopic resection plus progestin. After a median follow up of three years, 74% achieved a complete response with a recurrence rate of 41%. Only 58% attempted to conceive, achieving a live birth rate of 17%. The conclusion was that this approach seemed to be feasible, although the population sample was very limited [78].

### 7.2. Lynch Syndrome

Lynch syndrome, or hereditary non-polyposis colon cancer (HNPCC,) is an autosomal dominant hereditary syndrome with high penetrance. It is caused by the mutation of a mismatch repair gene, involved in DNA mismatch repair (MMR)—MLH1 (34% of cases), MSH2 (51%), MSH6 (49%) and PMS2 (24%) [79,80].

Women with an MSH6 mutation are at increased risk of endometrial cancer (HR = 25.5, 95% CI = 16.8 to 38.7) [81].

Lynch syndrome accounts for approximately 3% of all ECs, but this figure rises to 9% of ECs in women under the age of 50 years [15,82,83,84,85].

Patients with Lynch syndrome have an increased risk of EC. In the general population the risk of EC is 2.5–3% [86], but in women older than 70 years with Lynch syndrome the risk of having EC is 39% [87].

To detect Lynch syndrome in patients, the following steps are recommended:

- The identification of susceptible patients from their personal and family histories. Amsterdam criteria (I and II) [88] have traditionally been used; however, they miss as many as 68% of patients. The Bethesda Guidelines were developed to provide broader clinical criteria for screening [89,90], but a considerable number of patients with Lynch syndrome are still not detected [91].
- The assessment of the reactive immunity for the mismatch repair genes (MLH1, MSH2, MSH6 and PMS2) in all endometrial malignant tumors in women under 70 years with a family or personal history of tumors associated with Lynch syndrome, with simultaneous tumors in the ovary, or when the tumor has suspicious microscopic features (e.g., high histological grade, intratumoral lymphocytes, location in the lower segment, etc.) [21].
- The performance of a genetic study in patients who meet all the Amsterdam criteria or any of the Bethesda criteria; as well as in patients with colorectal or endometrial cancer with evidence of DNA repair alteration or with a first or second degree relative with a known MRS mutation [92].

There is no definitive scientific evidence of the impact of routine screening for endometrial carcinoma in patients diagnosed with Lynch syndrome. Due to the increased risk of endometrial cancer in this group and the associated morbidity and mortality, it is agreed that systematic screening is indicated. Based on expert recommendations, a transvaginal ultrasound and/or endometrial biopsy should be performed annually from the age of 30–35 years onward [21,93].

A reduction in the incidence of endometrial cancer has been observed associated with prophylactic hysterectomy and bilateral annexectomy. However, there is no scientific evidence that risk-reducing surgery is associated with a decrease in mortality from these causes. The main guidelines state that the possibility of prophylactic surgery, hysterectomy and bilateral annexectomy should be evaluated, once the wish to give birth has been fulfilled [21].

Therapeutic management of endometrial cancer in patients with Lynch syndrome does not present any differences with respect to sporadic EC. There is no consensus on the conservative management of EC in Lynch syndrome patients. According to a European survey, Lynch syndrome is a contraindication for conservative management in half of the responders [94]. However, there is no general agreement. If conservative treatment is considered, it must comply with the standard to respect usual guidelines of EC management [95].

## 8. Conclusions

There is a consensus across the leading gynecologic oncology societies that fertility-sparing treatment is feasible and safe for young patients with G1 endometrioid EC limited to the endometrium. The use of progestins seems to achieve very good response rates. Combination treatments such as metformin or hysteroscopy resection following medical therapy may improve the recurrence rate. The molecular classification must be included to individualize the treatment. ARTs may shorten the time to conception.

The mortality with this approach is very low, despite the very high rate of recurrence. The overall survival at 15-year follow up is around 90%. With the evidence provided by the published meta-analyses, we observed a complete response rate around 75% for oral progestin, and around 79% for LNG-IUS. The live birth rate was between 20–48%, depending on the treatment performed or the use of ART. The recurrence rate was close to 33% and was lower in studies that used LNG-IUS (11%) or hysteroscopic resection (14%).

The addition of metformin to progestogens, hysteroscopic resection of AEH and EC associated with LNG-IUS or other treatments; weight reduction with bariatric surgery or other interventions should be studied in more detail. All these treatments, alone or in combination, are options for the future.

In the absence of larger prospective studies, it is very important to consider overall health and fertility potential prior to recommending non-standard oncologic treatments. Further randomized controlled trials are now needed to offer stronger evidence.

## Figures and Tables

**Table 1:** Optimal indications for fertility-sparing treatment.

1	Histologically confirmed endometrioid type endometrial adenocarcinoma
2	Well-differentiated tumor
3	Disease confined to the endometrium
4	No evidence of myometrial invasion on imaging study
5	No clinical evidence of extrauterine disease
6	Strong desire to preserve fertility
7	Age < 40 years (ideally)
8	No contraindication to medical treatment
9	Informed consent, expanding that this is not a standard treatment and carries a higher risk of recurrence

Adapted from [24].

**Table 2:** Features of the four molecular subtypes. Adapted from [32,33].

Subtype	POLE-Mutant	MMRd (MSI)	CN Low (p53 wt)	CN High (p53 Abn)
Somatic copy-number alterations	Very low	Low	Low	High
Top five recurrent gene mutations	POLE (100%)	PTEN (88%)	PTEN (77%)	TP53 (92%)
	PTEN (94%)	ARID1A (37%)	PIK3CA (53%)	PIK3CA (47%)
	DMD (100%)	PIK3CA (54%)	CTNNB1 (52%)	FBXW7 (22%)
	CSMDI (100%)	PIK3R1 (42%)	ARID1A (42%)	PPP2R1A (22%)
	FAT4 (100%)	RPL22 (37%)	PIK3R1 (33%)	PTEN (10%)
Associated histological features	Endometrioid Grade 3 Ambiguous morphology	Endometrioid Grade 3 LVSI substantial	Endometrioid Grade 1–2 ER/PR expression	Serous Grade 3 LVSI
Associated clinical features	Lower BMI Early Stage (IA/IB)	Higher BMI Lynch Syndrome	Higher BMI	Lower BMI Advanced Stage
Prognosis in early stage	Excellent	Intermediate	Excellent, Intermediate	Poor
Diagnostic test	Sanger/NGS	MMR-IHC: MLH1, MSH2, MSH6, PMS2 MSI assay		P53-IHC NGS

Abbreviations: BMI, body mass index; CN, copy-number; ER, estrogen receptor; IHC, immunohistochemistry; LVSI, Lymph-vascular space invasion; MMRd, mismatch repair deficiency; MSI, Microsatellite instability; NGS, next-generation sequencing; PR, progesterone receptor.

**Table 3:** Summary of Systematic reviews and Meta-analyses of Fertility-Sparing Treatments.

Author Year	Outcomes	N° Studies	Intervention	Complete Response (%) (95% CI)	Relapse (%) (95% CI)	Pregnancy Rate (95% CI)	Live Birth Rate (95% CI)	Follow-Up (Months) Mean (Max-Min)
Gallos et al., 2012 [43]	Regression Relapse Live birth rate	38 studies 408 EEC 151 CAH Age: N/R	OP LNG-IUS Hysteroscopy	76.2 (68–85.3) *	40.6 (33.1–49.8) **	N/R	28 (21.6, 36.3)	11–76.5
Baker et al., 2012 [45]	Complete response Relapse	12 studies 219 OP (117 CAH 102 EEC) Age: 19–77 years 11 studies LNG-IUS (EC) Age: N/R	OP LNG-IUS	CAH 74 (65–81) EC 72 (62–80) EC68 (45–86)	20.1	N/R	N/R	Mean 45.8 6–71
Koskas et al., 2014 [69]	Remission (12 m) Remission (24 m) Recurrence (12 m) Recurrence (24 m) Pregnancy rate (22 studies, 351 w)	24 studies 370 women (AEH/EC) Age: 19–44 years	MA MPA Other ***	78 81.4	9.6 29.2	32	N/R	Mean 48.86
Wei et al., 2017 [45]	Complete response Relapse response Pregnancy rate Live birth rate	28 studies 1038 women (CAH/EEC) Age: 27.5–57.5 years	OP LNG-IUS	71 (63–77) 76 (67–83)	29 (19–40) 9 (5–17)	34 (30–38) (18 studies) 18 (7–37) (two studies)	20 (16–25) (11 studies) 14 (9–23) (two studies)	Mean 40.6
Luo et al., 2018 [70]	Regression rate	1 RCT 19 patients CAH Age: N/R	OP LNG-IUS	77 100 2.76 (0.26–29.73) ^+^	N/R	N/R	N/R	Mean 6
Fan et al., 2018 [71]	Complete response Recurrence rate Pregnancy rate	28 studies 619 women EEC (1) OP (456 w) (2) Hysteroscopy ^+^ Progestin (73 w) (3) LNG-IUS plus Progestin (90 w) Age: <45 years	OP HR + PT LNG-IUS ^+^ GnRH-a/ Progestin	76 (70–81) 95 (87–100) 72.9 (60–82)	30 (21–42) 14 (7–26) 11 (5–22)	52 (41–66) 47.8 (33–69) 56 (37–73)	N/R	Mean 41.3
Guillon et al., 2019 [72]	Remission rate Prognostic factors	65 studies 1604 women AEH/EEC Age: Mean 32.1 years	MA MPA LNG-IUS Other ***	0.75 (0.73–0.77) ^+^	N/R	N/R	N/R	Mean 34.7
Chae-Kim et al., 2021 [73]	Relapse rate Regression rate Pregnancy Live birth rate	6 studies 621 women AEH/EEC Progestin + metformin (241 w) Age: Mean 33.8 years Progestin (380 w) Age: Mean 34.6 years	Progestin ^+^ metformin Progestin	1.35 (0.91–2.00) ^++^ *p* = 0.14	0.46 (0.24–0.91^) ++^ *p* = 0.003	1.01 (0.44–2.35) ^++^ *p* = 0.98	0.46 (0.21–1.03) ^++^ *p* = 0.06	Mean 28.7

Abbreviations: CAH, complex atypical hyperplasia; AEH atypical endometrial hyperplasia; EC, endometrial cancer; EEC, early endometrial cancer; CR, complete response; RR, relapse response; PR, pregnancy rate; LNG-IUS, levonorgestrel-releasing intrauterine-system; GnRH-a, gonadotropin-releasing hormone agonist; HR + PT, hysteroscopic resection + progestin therapy; N/R, Not reported; OR, odds ratio; OP, oral progestin. * Regression rate (95% CI). ** Relapse rate (95% CI). *** Oral contraceptives, other progestogens, GnRH-a, LNG-IUS and induction of ovulation or GnRH-a. ^+^ Comparison of OP and LNG-IUS; OR (95% CI). ^++^ Comparison of progestin + metformin vs. progestin; OR (95% CI).
